# Manual Diseases of the Skin

**Published:** 1854-02

**Authors:** 


### EDITORIAL.

*Manual of Diseases of the Skin*, from the French of M. M. CAZENAVE and SCHEDEL, with notes and additions, by THOS. H. BURGESS, M. D., etc. Second American edition, enlarged and corrected from the last French edition, with additional notes, by H. D. BULKLEY, M. D., etc. New York: Samuel S. & W. Wood, 1852.

THE researches of M. M. Cazenave and Schedel, have added much to our knowledge of this difficult branch of pathology. Following the excellent classification of Willan, as modified by Biett, they have described in a clear and graphic manner the various diseases incident to the skin. Our knowledge of the anatomical lesions in these diseases, has been so imperfect, and the difficulties to be overcome in acquiring that knowledge so numerous, that a natural classification based on the structures affected, and arranged with reference to the special pathology of these structures, is a thing almost impossible. The classification of Wilson is perhaps the most perfect attempt at such an attainment. His four general classes comprise

I. Diseases of the Derm.

II. Diseases of Sudoriparous glands.

III. Diseases of the Sebiparous glands.

IV. Diseases of the hairs and the hair follicles.

Each of these classes is divided into genera and species, the whole constituting a most beautiful arrangement for the pathologist, but of far less practical value than that adopted by our authors. In making a diagnosis of diseases of the skin, we must be guided by sight, and in order to discriminate between them we must seize upon those characters uniformly present in individuals of the class and absent in others; and which serve to define its species, while in most instances they also indicate its pathology.

The present edition contains not only the last opinions of the authors, but also the valuable notes of the translator and the American Editor.

In the article on *Scabies*, which has been entirely re-written by the authors in the last French edition, we find the following in reference to the insect producing the disease:

M. Albin Gras has studied the action of certain articles on it to ascertain what will destroy it the soonest. He found that it lived three hours in pure water; two hours in olive oil; one hour in a solution of sugar of lead; three quarters of an hour in lime water; twenty minutes in vinegar, alcohol, and an alkaline solution; twelve minutes in a solution of sulphuret of potassa; nine minutes in spirits of turpentine; from four to six minutes in a concentrated solution of hydriodate of potassa. It survived one hour in the vapor of sulphur under a watch glass. The solution of the hydriodate of potassa is therefore the most powerful agent which can be used externally with safety. M. Albin Gras has extracted them alive from a patient who had taken three sulphur baths, while he has frequently found them destroyed after a single friction with the ointment of Helmerich.

Scabies is never caused by inoculation with the fluid from the vesicles, nor by that from any other of the eruptions produced by the insect, but only by the insect itself.

The acarus scabiei is a small, round, greyish body, sometimes in motion, and at other times at rest. With good sight, and especially with a magnifying glass, the head and fore legs of the insect can be easily distinguished. Under the microscope, it presents an oval body, the back somewhat convex, on which are marked numerous fine curved lines, parallel with each other, and of unequal length in the different groups. Under the abdomen are eight legs, four of which are in front, and have at their extremities a sucker of trumpet shape, with hairs at their base. The four hind claws are without suckers, and terminate with a hair of greater or less length. The head is covered with fine hairs, and has a trumpet-shaped protuberance, shorter then those on the fore legs, with a hair longer than itself on each side of it.*

It is believed by our authors that the acarus is the sale cause of itch, that it can be communicated from a dead subject so long as the insect is alive.

With a view of destroying the parasite common salt, corrosive sublimate, arsenic, salts of copper, iron and zinc, a decoction of the leaves of tobacco, hyosciamus or hellebore, have been recommended.

---

*For a scientific and much more minute description of the acarus scabiei, see *Wilson on Diseases of the Skin*, second edition, 1847. H. D. B.

Our readers will remember that a previous volume of this journal contains a short notice of Dr. Hardy's treatment, by which the time required for a cure has been reduced to two hours.

The original essay of Dr. Burgess on Glanders & Farcy is retained, although the authors themselves in the last French edition have introduced a chapter on that subject.

A case of this disease was reported in the *N. York Journal of Medicine and the Collateral Sciences* for Sept. 1853, in the person of the late Dr. Stoutenburgh of L. .I

The premonitory symptoms were those of a cold, headache, loss of strength ,pain in the joints, &c. During the progress of the disease there was observed a thick yellow coat on the tongue, pain in chest, a sense of stricture, bowels constipated, increased temperature of the surface, pulse 90, moderately full, respiration hurried, restlessness, prostration of the vital powers with a typhoid tendency, urine scanty and of a blood color ; near the termination of the disease there appeared on different parts of the body and imbedded in the cellular tissue, indurated patches, elevated above the surface and rapidly tending to ulceration. Finally, with an aggravation of all the preceding symptoms, spasmodic action of the glottis, great difficulty of respiration and delirium. There was evidently previous to death a great tendency to organic dissolution.

In this case the disease manifested its specific characters only a short time previous to death in the form of Farcy buds, as they are called.

In acute glanders uncomplicated with Farcy, in addition to the promonitory symptoms already described, Dr. Burgess has enumerated the following :

The patient complains of great heat about the nose and windpipe, accompanied with a copious viscid discharge, and with pain in the head, back, and limbs, and constriction about the chest. After a certain period, the nose and surround ng parts become swollen, hot, excoriated, and of a bright red or l vid color : one or both eyes are inflamed, or completely closed ; a profuse tenacious mucus, at first of a deep yellow, but afterwards of a bloody or dark sanious appearance, exudes from the nostrils, and occasionally from the eyes ; hard, round, phlyzа-

cious pustules appear on different parts of the body; the temperature of the skin is increased; the pulse is rapid, soft, and weak or undulating; respiration quick, weak and shallow. The tongue dry, rough, and reddish brown; the body is bathed in copious and offensive perspiration, the thirst unquenchable, the stools are slimy, and horribly fœted, the voice is weak, and the mind wandering. In the course of a few days these symptoms become still more aggravated; diffused abscesses appear in various parts of the body, especially about the joints. The fever assumes a more malignant character; the disease extends to the air-passages and lungs; fresh abscesses form and suppurate; the nose and surrounding parts become gangrenous; the perspiration is more profuse and sour; finally, a state of general collapse ensues, and death is ushered in by a low muttering delirium; the fœtor from the discharges, and from the whole body, towards the close of the disease, is insupportable.

The prognosis is always unfavorable.

We are not acquainted with any method of treatment that promises success, but we certainly should not prescribe for a patient laboring under the symptoms present in the case of Dr. Stoutenburgh under the care of Dr. Strew, calomel and antimony.

Since writing the above we have read with interest a notice of two cases of glanders cured by the administration of sesquicarbonate of ammonia in large doses, frequently repeated and conjoined with wine and tonics.

The cases were originally published in the *London Journal of Medicine* and are embodied in the report on practical Medicine contained in the last No. of Ranking's abstract.

The work is a valuable one and can not well be dispensed with by those who would make themselves familiar with the diagnosis, pathology and treatment of cutaneous affections.

For sale by A. H. & C. Burley. J.

